# Calendar

**DOI:** 10.1155/S1463924681000606

**Published:** 1981


### Results and discussion

The system described satisfies to the following requirements:

1. Deviation from desired temperature is ±0.01 K.
2. Rapid attainment of desired temperature. When working at low temperatures at least a factor of 100 faster than conventional thermostats.
3. Temperature programming is easily performed.
4. Does not introduce noise into the measuring system.

The time necessary to reach the desired temperature within ±0.01 K depends on the magnitude of the change of temperature. A change of 10 K requires ten minutes, while a larger change, eg 20 K, can be performed within 15 to 20 minutes. An example is shown in Figure 3.

According to the specification of the Peltier circuits, the maximum temperature difference that can be obtained between the two sides of the circuit is approximately 60 K, ie the temperature of the thermostat can vary within ±60 K from the temperature of the coolant medium. The reason is that at 60 K the normal heat transfer through the Peltier elements due to the temperature difference is equal to the heat transfer produced by the Peltier effect (see Figure 4). Thus the net heat pump effect is zero when the temperature difference between the hot and cold sides is 60 K. To put it more generally, the maximum temperature difference that can be obtained depends on how well isolated the system is. It should be pointed out that the upper limit is set by the fact that the circuits are not designed to resist temperatures higher than +70°C (90°C for short periods). Since this system uses tap water of 15-20°C as coolant, and since it is not well isolated, the temperature range is roughly -30 to +70°C.

Most conventional thermostats use alternating current and generally they are controlled in such a way that all of the heating current is switched on and off. Such a system is a possible source of noise, which might disturb the measuring system. In the case of potentiometric titrations where high impedence electrodes are used, care must be taken to shield the measuring system from the noise generated by the thermostat. With the approach described here, this source of noise is eliminated. The system is driven by direct current, and when the desired temperature is attained, only very small changes of current are needed to maintain the temperature constant.

REFERENCES

[1] Light, T.S. in "Ion-Selective Electrodes" Ed Durst, R.A., 1969, Nat Bur Stand (US), p 354.

[2] Bates, R.G. in "Determination of pH", 1973, Wiley.

### Calendar

**Editor's Note:**
*Organisers of conferences, seminars etc. should send details for inclusion in this calendar as soon as the relevant information is available and not later than three months before the event.*

**Chemistry and analysis of hydrocarbons in the environment, workshop**
November 26-27, Barcelona
*J. Albaiges, Expoquimia, Plaza de Espana, Barcelona-4, Spain*

**In-stream and on-line particle size analysis**

December 2, London

*Dr N.G. Stanley-Wook, University of Bradford, Bradford*

**Capillary columns**
December 3, London
*Miss P.E. Hutchinson, Royal Society of Chemistry, Burlington House, London W1*

**Using microcomputers and microprocessors in the laboratory**
December 4, Chepstow
*Miss P.E. Hutchinson, Royal Society of Chemistry, Burlington House, London W1*

**Computer aided chemistry, specifically related to chromatography and spectroscopy**
December 7-8, London.
*Dr B.P. Chadburn, Perkin-Elmer Ltd, Post Office Lane, Beaconsfield, Bucks*

**Recent advances in analytical chemistry**
December 9-11, London
*Executive Secretary, The Royal Society, 6 Carlton House Terrace, London, SW1Y 5AG*

**Photoacoustic imaging**
December 10, London
*Dr B.C. Beadle, Scientific Conference Services Ltd, 14 Trading Estate Road, London, NW10 7LU*

**Computer aided chemistry, specifically related to chomatography and spectroscopy**
December 10-11, Manchester
*Dr. B.P. Chadburn, Perkin-Elmer Ltd, Post Office Lane, Beaconsfield, Bucks*

**Atomic absorption and emission spectrometry, course**
December 14-18, Loughborough
*Dr J.F. Tyson, Dept of Chemistry, University of Technology, Loughborough Leics, LE11 3TU*

**Moisture measurement seminars**
December 19 & January 13, Luton
*Anachem Ltd, 15 Power Court, Luton, Beds, LU1 3JJ*

**1982**

**Plasma spectrochemistry, 1982 Winter Conference**
January 4-9, Florida
*Conference Chairman, c/o ICP Information Newsletter, Dept of Chemistry, GRC Towers, University of Massachusetts, Amherst, Mass 01003, USA*

**Microprocessor familiarisation course**
January 25-26, Sevenoaks
*Frankie Kingston, Sira Institute Ltd, South Hill, Chislehurst, Kent BR7 5EH*

**The Pittsburgh Conference,**
March 8-12, Atlantic City,
*Pittsburgh Conference, Department J-168, 437 Donald Road, Pittsburgh, PA 15235, USA*

**12th Annual Symposium on the Analytical Chemistry of Pollutants**
April 14-16, Amsterdam.
*Prof Dr. R.W. Frei, Congress Office, 12th Annual Symposium on the Analytical Chemistry of Pollutants, Congress Bureau, Vrije Universiteit, PO Box 7161, 1007 MC Amsterdam, The Netherlands.*

**International Congress on Automation in the Clinical Laboratory**
April 19-22, Barcelona, Spain.
*Dr. R. Galimany, Departmento de Analisis Clinicos, Seccion de Automatizacion, Cuidad Sanitaria 'Principes de Espana', Hospitalet de Llobrogat, Barcelona, Spain.*

**Analytica '82**
April 27-30, Munich
*ECL Exhibition Agencies Ltd, 11 Manchester Square, London W1M 5AB*

Abstracts continued from page 171

### Page 198

**An automatic titration system**
T. Pap *et al*

A titration system controlled by a desk top calculator was prepared. The volume fractions of the titrant were controlled using an exponential function using the calculator. The titration data are stored and the curve recorded. A titration of the mixture of formic and acetic acids and the evaluation process using an iterative method are presented.

**Un systèm de titration automatique**

Un système de titration contrôlé par une calculatrice de table a été construit et décrit. Les increments du réactif titrant sont determinés par lar calculatrice à l'aide d'une fonction exponentielle. Les données de la titration sont memorisées et la courbe tracée. Le dosage d'un melange de l'acide formique et de l'acide acétique et du procès de l'evaluation pour laquelle une methode itératif est appliquée sont decrits.

**Ein automatisches Titriersystem**

Ein durch einen Tischrechner gesteuertes Titriersystem wurde entwickelt und ist hier beschrieben. Die Volumeninkremente des Titriermittels wurden mit einer Exponentialfunktion im Rechner festgelegt. Die Titrationsdaten werden abgespeichert und die Messkurve aufgenommen. Die Titration eine Mischung von Ameisen- und Essigsäure und des Auswerteprozesses, der eine iterative Methode benützt, wurden dargelegt.

### Page 201

**An evaluation of the Technicon Star System for the estimation of serum $T_4$ and $T_3$ uptake**
S.G. Welshman *et al*

A Technicon Star System is evaluated for the routine R.I.A. estimation of serum $T_4$ and $T_3$-uptake. The within-batch and between-batch precision for $T_4$ and $T_3$-uptake were considered to be satisfactory. There was no significant carry-over or drift. The recovery of $T_4$ was excellent and there was a close correlation with the manual Amersham (PEG) assay. The Star analyser estimated samples at the rate of 60 per hour and was capable of processing a daily work-load of 200 $T_4$ and $T_3$-uptake assays. Over a trial period of nine months the system was virtually trouble-free and resulted in a significant reduction in man-power which is normally required for these tests.

**Evaluation du système Technicon Star pour la détermination de l'absorption du sérum $T_4$ et $T_3$**

Un système Technicon Star est évalué pour la détermination de routine R.I.A. de l'absorption du sérum $T_4$ et $T_3$. La précision dans la même série et entre séries etait considéré satisfaisante. Les valeurs retrouvées du $T_4$ etaient excellentes et la corrélation avec la méthode manuelle Amersham (PEG) était bonne. Le système Star permettait d'analyser 60 échantillons par heure et était capable d'éffectuer 200 analyses journalieres d'absorption $T_4$ et $T_3$. Pendant une période d'essay de neuf mois le système n'a jamais donnés de problèmes et a permis de réduire d'une façon significative le personnel nécessaire pour ces analyses.

**Eine Beurteilung des Technicon Star Systems für die Abschätzung der Aufnahme von Serum $T_4$ und $T_3$**

Ein Technicon Star System wird beurteilt im Bezug für die routinemässige R.I.A. Abschätzung der Aufnahme von Serum $T_4$ und $T_3$. Die Präzision innerhalb einer Serie und von Serie zu Serie für die $T_4$ und $T_3$ Aufnahme werden als genügend angesehen. Es wurde keine signifikante Verschleppung oder Drift beobachtet. Die Erholung von $T_4$ war ausgezeichnet und die Korrelation mit dem manuellen Amersham (PEG) Test war eng. Der Star Analysator bearbeitete Proben mit einer Geschwindigkeit von 60 pro Stunde und war befähigt, die tägliche Arbeitslast von 200 $T_4$ und $T_3$-Aufnahmetests zu ürbernehmen. Während einer Versuchsperiode von 9 Monanten zeigten sich faktisch keine Schwierigkeiten. Daraus resultierte eine signifikante Reduktion an Arbeitsstunden, die normalerweise für solche Bestimmungen erforderlich sind.

### Page 203

**Evaluation of the biochemical analyser Olli C + D after over a year of use in enzymology**
G. Baraton *et al*

An evaluation of Olli C + D system carried out according to the rules suggested by the French Society for Clinical Biology (SFBC) is presented. Data on optical performances (precision, linearity) of C system, accuracy (within run and day to day) of dilutor D system, and control of thermostatisation were given. Biochemical performances were tested for routine enzymologic determinations by within and between day precision and day to day precision over one year period.

**Evaluation de l'analyseur biochimique OLLI C+D après plus d'un an en enzymologie**

Une évaluation du système OLLI C+D a été effectuée selon les règles suggérées par la Societé Française pour Biologie Clinique (SFBC). Les performances optiques (précision, linéarité) du système C, L'exactitude (d'une série et de jour en jour) du système de dilution D et le contrôle de thermostatisation sont présentés. Les performances biochimiques ont été testées par des déterminations enzymologiques de routine pendant une année en étudiant la précision le même jour, entre deux jours et de jour en jour.

**Beurteilung des biochemischen Analysators OLLI C+D nach einem Jahr Einstaz in der Enzymologie**

Es wird eine Evaluation des OLLI C+D Systems vorgestellt, die gemäss der Regeln, die von der Französischen Gesellschaft für klinische Biologie (SFBC) vorgeschlagen sind, durchgeführt wurde. Daten werden angeführt für die optischen Leistungen (Präzision, Linearität) des C Systems, für die Genauigkeit (innerhalb einer Serie und von Tag-zu-Tag) des D Verdünnungssystems, und für die Thermostatisierung. Biochemische Leistungen wurden für routinemässige enzymologische Bestimmungen geprüft durch Beobachtung der Präzision innerhalb eines Tages und von Tag-zu-Tag für über ein Jahr.

### Page 206

**A microcomputer controlled thermostat**
A. Granéli *et al*

A microcomputer-controlled thermostat system based on commerciably available Peltier elements is described. The temperature can be changed rapidly. For 100 ml of aqueous solution, the desired temperature ± 0.01 K is attained within 10 - 20 minutes. The temperature range is -30 to +70°C.

**Un thermostat contrôlé par microordinateur**

Un thermostat contrôlé par microordinateur, se basant sur des éléments Peltier commerciaux, est décrit. La température peut être changée rapidement. Pour 100 ml de solution aqueuse, la température désirée ± 0.01K est atteinte en 10 - 20 minutes. La plage de température permise est de -30 à + 70°C.

**Thermostat mit Mikrocomputer-Steuerung**

Ein Thermostat mit Mikrocomputer-Steuerung wird beschrieben, der auf kommerziell erhältlichen Peltier Elementen basiert. Die Temperatur kann sehr rasch geändert werden. Für 100 ml einer wässrigen Lösung wird die gewünschte Temperatur ± 0,01 K innerhalb von 10 bis 20 Minuten erreicht. Der Temperaturbereich ist – 30 bis + 70°C.

